# Synthesis Mechanism of Low-Voltage Praseodymium Oxide Doped Zinc Oxide Varistor Ceramics Prepared Through Modified Citrate Gel Coating

**DOI:** 10.3390/ijms13045278

**Published:** 2012-04-24

**Authors:** Wan Rafizah Wan Abdullah, Azmi Zakaria, Mohd Sabri Mohd Ghazali

**Affiliations:** 1 Advanced Materials and Nanotechnology Laboratory, Institute of Advanced Technology, Universiti Putra Malaysia, 43400 UPM Serdang, Selangor, Malaysia; E-Mail: wanrafizah@umt.edu.my; 2 Department of Physics, Faculty of Science, Universiti Putra Malaysia, 43400 UPM Serdang, Selangor, Malaysia; E-Mail: mgm.sabri@gmail.com

**Keywords:** citrate gel, praseodymium oxide, varistors, zinc oxide

## Abstract

High demands on low-voltage electronics have increased the need for zinc oxide (ZnO) varistors with fast response, highly non-linear current-voltage characteristics and energy absorption capabilities at low breakdown voltage. However, trade-off between breakdown voltage and grain size poses a critical bottle-neck in the production of low-voltage varistors. The present study highlights the synthesis mechanism for obtaining praseodymium oxide (Pr_6_O_11_) based ZnO varistor ceramics having breakdown voltages of 2.8 to 13.3 V/mm through employment of direct modified citrate gel coating technique. Precursor powder and its ceramics were examined by means of TG/DTG, FTIR, XRD and FESEM analyses. The electrical properties as a function of Pr_6_O_11_ addition were analyzed on the basis of *I-V* characteristic measurement. The breakdown voltage could be adjusted from 0.01 to 0.06 V per grain boundary by controlling the amount of Pr_6_O_11_ from 0.2 to 0.8 mol%, without alteration of the grain size. The non-linearity coefficient, α, varied from 3.0 to 3.5 and the barrier height ranged from 0.56 to 0.64 eV. Breakdown voltage and α lowering with increasing Pr_6_O_11_ content were associated to reduction in the barrier height caused by variation in O vacancies at grain boundary.

## 1. Introduction

Applications of low-voltage varistors for circuit protection are increasingly significant due to growing demands on low-voltage electronics. For instance, battery powered and mobile appliances require protection from transient voltage of between 4 to 20 V (dc voltage) while many communication devices need fast response protection from transient voltage of 22 –68.V [1–3]. These factors create needs for continuous development of ZnO varistor materials with fast response, highly non-linear current-voltage characteristics and energy absorption capabilities at low breakdown voltage.

Meanwhile, trade-offs between breakdown voltage, *E_b_*, and grain size, *d*, in conventional bismuth (Bi_2_O_3_) based ZnO ceramics poses a critical bottle-neck in production of low-voltage varistors. This is because the effective breakdown voltage of a varistor is proportionate to the number of grain boundaries per unit thickness and the inverse to grain size. Consequently, lowering the breakdown voltage in Bi_2_O_3_-ZnO varistor having average breakdown voltage per individual grain boundary, *V_gb_*, of approximately 3.2–3.5 V is a great challenge [4–6].

Most existing preparation techniques rely strongly on ZnO grain manipulation processes. The most classical ways of making low-voltage varistors are through grain coarsening techniques by making varistor from crushed ceramics, prolonged sintering processes at higher temperatures and adding grain growth enhancers such as TiO_2_ [7]. Other ways include employment of seeding technique by using grown ZnO crystal seeds as precursor [8–10] and deposition technique to fabricate multilayered thin film varistors with interdigitated electrodes [11]. Grain coarsening and seeding techniques could be economically less feasible as they are energy intensive processes and suffer from inhomogeneous microstructure which leads to inconsistent current-voltage characteristics [8]. Multilayered thin film varistors obtained from deposition technique on the other hands, are lingered with structural integrity issues [11]. The present work has discovered that it is possible to obtain low-voltage varistors made of praseodymium (Pr_6_O_11_) based ZnO ceramics through employment of a solution coating technique known as direct modified citrate gel coating. The technique involves coating of additive precursor in citrate gel form onto fine ZnO particles and proceeds with calcination and sintering processes. Similar methods using different starting materials, medium and deposition sequences have been adopted previously in [12–14] for obtaining both low and high-voltage varistors but limited to Bi_2_O_3_–ZnO systems. Advantages of this preparation approach are for example:

- Offer direct chemical route to prepare metal oxide ceramics at reduced sintering temperature and time in comparison to conventional solid state route or several chemical techniques such as sol-gel and co-precipitation [12–15].
- Ability to control the homogeneity of solutions particularly during preparation of multi-components ceramic materials through prevention of side precipitation or sedimentation [14,15].
- Improve distribution of dopants and facilitate diffusion of additives on ZnO grains by encouraging contact during precursor preparation [14].
- Improve grain size consistency by controlling the formation of ultrafine grains and abnormal grain growth [13].

Pr_6_O_11_ based ZnO ceramics are the materials of interest for this study. Based on the recent trend, Pr_6_O_11_ based ZnO ceramics have been actively researched to overcome drawbacks in existing Bi_2_O_3_ based ZnO varistor materials such as Bi_2_O_3_ vaporization and formation of Bi-containing secondary phases when sintered at temperatures over 1000 °C [16–20]. Ramirez *et al.*, [16] and Furtado *et al.*, [17] demonstrated that Pr_6_O_11_ improved effective electrical current flow by restraining the formation of secondary phases and induced densification of varistor during fabrication. Zhu *et al.*, [18] claimed that Pr_6_O_11_ exhibited grain growth suppressing effect that controlled the overall development of grain during sintering. Thus, their studied varistor ceramics had more uniform and compact microstructures. Several series of high density and high stability Pr_6_O_11_ based ZnO varistor ceramic systems containing more than four combination of rare earth and transition metal oxides have been developed and reported in Nahm [19,20]. The proposed varistors exhibited comparable non-linearity properties to that of Bi_2_O_3_ based ZnO varistors with minimum number of additives. The non-linearity coefficient of the varistors could reach up to 60 with the general *V_gb_* of 2–3 V. However, most of the Pr_6_O_11_ based ZnO varistor systems that have been reported so far are developed towards high-voltage applications. The work by Horio *et al.*, [21] is one of the very few attempts to extend the use of Pr_6_O_11_ based ZnO ceramics for low-voltage applications. They have successfully fabricated ZnO/Pr_6_O_11_ multilayered thin films having the non-linearity coefficient, α of 10 and *E_b_* of 20 V by the radio-frequency (RF) sputtering in Ar/O_2_ environment. Hence, further research is needed in order to take advantage of these potentially high non-linearity and high stability ceramic materials for satisfying low-voltage requirements.

The purposes of this study are to develop Pr_6_O_11_ based ZnO varistors by modified citrate gel coating technique for low-voltage applications and comprehensively investigate synthesis mechanisms involved throughout the preparation steps. In this work, a single-doped system containing ZnO and varied Pr_6_O_11_ content has been prepared. The precursor powder was studied with various methods including ATR-FTIR spectroscopy, TG-DTG, XRD and FESEM analyses. Meanwhile, the varistor ceramics were characterized using XRD and FESEM. The electrical properties of the ceramics were discussed on the basis of *I-V* characteristic measurement.

## 2. Results and Discussion

### 2.1. Chemical, Thermal and Microstructure Analysis of Precursor Powder

In agreement with the work by Lorenz *et al.*, [14], Pr(III) acetate salt reacted with one of the three carboxylic groups in citric acid to produce Pr(III) citrate complexes according to the following reaction;

(1) $$3({\text{C}}_{6}{\text{H}}_{5}{{\text{O}}_{7}}^{-})+{\text{Pr}}^{3+}+3{\text{H}}^{+}\overset{60-70{}^{\circ}C}{\to }{\text{Pr}}^{3+}{\left[{\text{C}}_{6}{\text{H}}_{5}{{\text{O}}_{7}}^{-}\right]}_{3}+3{\text{H}}^{+}$$

The uncoordinated carboxylic chains chelated on the ZnO particles dispersing in the solution to form firmed citrate gel coating layer. The layer was thermally converted during calcination process at 500 °C for 4 h and sintering process at 1200 °C for 1 h according to the following oxidation reaction.

(2) $$2\;\text{Pr}({\text{C}}_{6}{\text{H}}_{5}{\text{O}}_{7})+\frac{49}{2}{\text{O}}_{2}\overset{500{}^{\circ}\text{C},4\;\text{h in Air}}{\to }\text{Pr}{\text{CO}}_{3}\text{OH}+34\;{\text{CO}}_{2}+15\;{\text{H}}_{2}\text{O}$$

(3) $$6\;\text{Pr}{\text{CO}}_{3}\text{OH}+\frac{13}{2}{\text{O}}_{2}\overset{1200{}^{\circ}\text{C},1\;\text{h in Air}}{\to }{\text{Pr}}_{6}{\text{O}}_{11}+6\;{\text{CO}}_{2}+3\;{\text{H}}_{2}\text{O}$$

Complete formation of Pr_6_O_11_ from PrCO_3_OH intermediate was achieved during sintering at 1200 °C. The synthesis mechanism was confirmed by Attenuated Total Reflectance Fourier Transform Infrared Spectroscopy (ATR-FTIR), Thermogravimetry/Differential Thermogravimetry (TG/DTG) and X-ray Diffractometer (XRD) analyses. The morphology of precursor powder was observed under Field Emission Scanning Electron Microscopy (FESEM).

#### 2.1.1. ATR-FTIR

**Figure 1:**
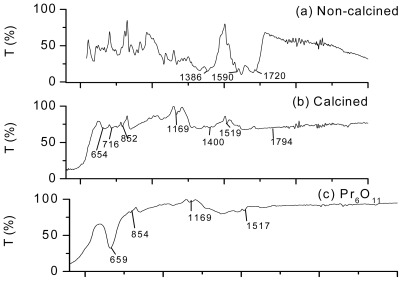

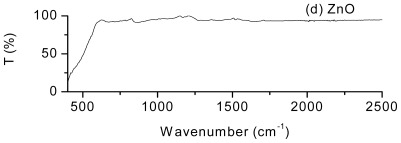
IR spectra of (**a**) ZnO powder coated with Pr citrate gel before calcination and (**b**) ZnO powder coated with Pr citrate gel after calcination in comparison to (**c**) Pr_6_O_11_ and (**d**) ZnO.

IR spectra of both non-calcined precursor powder in Figure 1a and calcined powder in Figure 1b were compared to spectra of pure Pr_6_O_11_ (Figure 1c) as well as ZnO (Figure 1d). It was found that the non-calcined precursor powder contained ZnO and Pr complexes. The asymmetrical and symmetrical stretching vibrations of carboxyl (–COO) in Pr citrate complexes was respectively observed at 1386 and 1590 cm^−1^. The result was consistent with [15]. The disappearance of several characteristic peaks in the region of 600–2500 cm^−1^ in the spectrum of calcined powder shown in Figure 1b were associated to elimination of carbonyl (C=O), carboxylate and alkyl groups at high temperature. The emergence of unidented carbonate group (-CO_3_) was supported by a new in-plane deformation vibration peak at 716 cm^−1^, asymmetrical stretching vibration peak at 1400 cm^−1^ and symmetrical vibration peak at 1794 cm^−1^ [22,23]. The presence of Pr-O lattice vibration mode at 655 cm^−1^ and other characteristic peaks of Pr_6_O_11_ (852, 1169 and 1519 cm^−1^) were also detected in the calcined powder spectrum [22]. Therefore, it is supported that Pr citrate gel layer coating the ZnO particles started to transform into Pr_6_O_11_ layer upon calcination.

#### 2.1.2. TG/DTG

**Figure 2:**
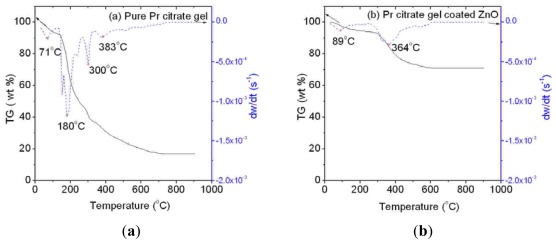
TG/DTG curves of (**a**) pure Pr citrate gel and (**b**) Pr citrate gel coated ZnO heated in air at heating rate of 10 °C/min.

TG/DTG curves in Figure 2 depict the decomposition profile of pure Pr citrate gel powder and coated ZnO precursor powder. Based on Figure 2a, the pure Pr citrate gel powder decomposed in four major steps as signified by derivative weight loss peaks at 71, 180, 300 and 383 °C, respectively. The first decomposition stage within the range of 32–139 °C was associated to the removal of physically trapped moisture. The subsequent process occurring between 140–266 °C was assigned to the rapid decomposition of excess citric acid. The citrates of Pr gel started to degrade within the temperature range of 266–334 °C to form stable phase of intermediate carbonates and/or oxides. Gradual and continuous weight loss beyond 334 °C was caused by slow decomposition of carbonates to oxides and overlapping decomposition of citrates in the previous stage. Figure 2b shows decomposition of coated ZnO precursor powder occurring in two stages respectively at 89 °C and 364 °C. The first stage was associated to simultaneous evaporation of moisture and excess citric acid. Meanwhile, the later was attributed to concurrent release of citrate anion and decomposition of carbonates. Decomposition trend of Pr citrate gel observed in this study was comparable to other metal citrate complexes reported in [22,23].

#### 2.1.3. XRD

**Figure 3:**
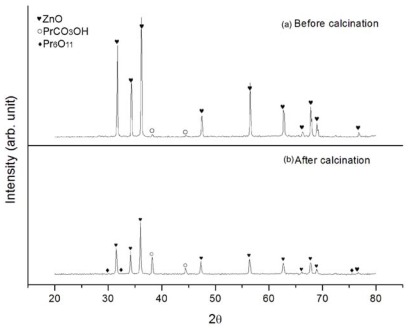
XRD patterns of (**a**) Pr citrate gel coated-ZnO before calcination and (**b**) Pr citrate gel coated-ZnO after calcination at 500 °C for 4 h.

XRD analysis of the calcination product in Figure 3 identified the presence of Pr carbonate hydroxide (PrCO_3_OH) phase (ICSD Reference code 00-026-1349) and traces of Pr_6_O_11_ phase (ICSD Reference code 00-042-1121) coexisting with hexagonal wurtzite ZnO phase (ICSD Reference code 01-075-0576). In comparison, initial powder consisted of mostly ZnO phase. Therefore, the phase analysis suggested that precursor powder after calcination contained mixture of ZnO, PrCO_3_OH and Pr_6_O_11_ phases.

#### 2.1.4. FESEM

**Figure 4:**
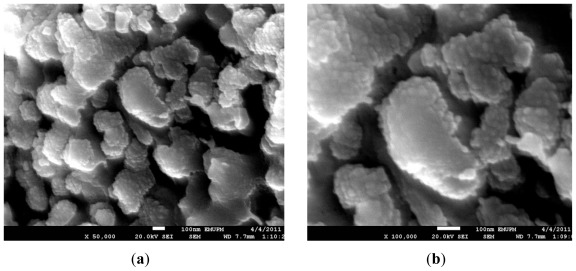
FESEM micrographs of precursor powder containing calcined ZnO and Pr dopant at (**a**) 50,000 and (**b**) 100,000 times magnification.

Figure 4 shows the morphology of precursor powder after calcination. The exterior of ZnO were adequately covered with layer of nano-scaled particles mostly PrCO_3_OH and Pr_6_O_11_ phases as confirmed in XRD analysis. The observation suggested that effective mixing of varistor precursor components has been achieved through optimal distribution of additive prior to sintering process. This technique simplified the process to achieve homogeneous mixture in comparison to typical demanding and time-consuming mechanical ball milling steps.

TG/DTG, ATR-FTIR, XRD and FESEM analyses suggested that ZnO particles have been successfully coated with Pr citrate gel. The gel was transformed to intermediate phase of Pr carbonates through calcination process. Partial cystallization of Pr oxide was also taken place during the calcination process.

### 2.2. Microstructure and Electrical Characteristics of Sintered Ceramics

#### 2.2.1. XRD

**Figure 5:**
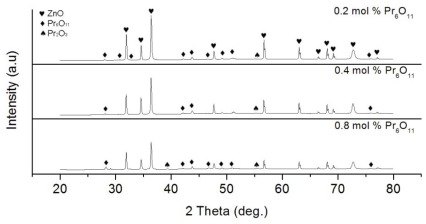
XRD patterns of sintered ZnO varistor ceramics with Pr_6_O_11_ content.

Figure 5 shows the XRD patterns of sintered ceramic sample comprising different Pr_6_O_11_ contents. The patterns confirmed the presence of dominant ZnO phase (ICSD Reference code 01-075-0576) with hexagonal wurtzite structure and cubic Pr_6_O_11_ phase (ICSD Reference code 00-042-1121) in all samples with no evidence of secondary phases. Traces of Pr_2_O_3_ (ICSD Reference code 00-022-0880) phase identified in samples were probably formed as a result of redox reaction in Pr_6_O_11_ at high temperature. Disappearance of peaks assigning to PrCO_3_OH phase suggested the complete conversion of Pr citrate gel layer on ZnO into Pr_6_O_11_.

#### 2.2.2. FESEM

**Figure 6:**
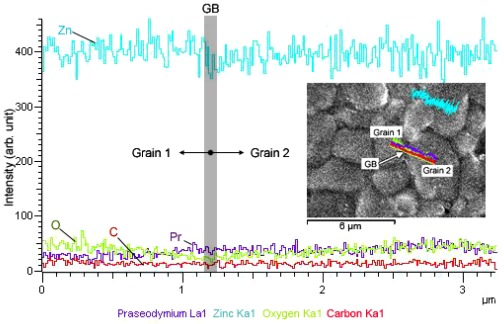
FESEM micrograph of the polished surface for sintered ceramic containing 0.8 mol % Pr_6_O_11_ and its corresponding EDAX line scan across two adjacent grains.

FESEM micrograph in Figure 6 represents the microstructure of sintered ceramic surface for ZnO varistor with 0.8 mol % Pr_6_O_11_. The bright spots on the ceramic surface indicated the area with heavier element like Pr. Spectra obtained from Energy Dispersive X-ray Spectroscopy (EDAX) line scan across two neighboring grains indicated that the concentration of Pr was increasing towards the grain boundary. Otherwise, the concentration of Zn and O were decreasing. This observation proposed that preferential distribution of Pr at grain boundaries was achieved and such microstructures are intended for formation of Schottky barrier that is responsible for the non-linearity behavior.

#### 2.2.3*. I-V* Characteristics

Table 1 shows the microstructure and non-linearity characteristic parameters of sintered ceramics. Pr_6_O_11_ doped ZnO varistors obtained from citrate gel coating technique had compact microstructures and were composed of fine grains. The average relative densities of ceramics exceeded 95% of theroretical density of bulk ZnO (5.61 g/cm^3^) while the average grain size was in the range of 4.5 to 5.2.μm. The result is consistent with [19,20]. *I-V* measurement revealed that *α*_1_ calculated from slope at low current region (10–100 mA/cm^2^) was in the range of 1.8–3.1. However, the α_2_ value measured at high current region (100–200 mA/cm^2^) ranged from 3.0 to 3.5. Both α_1_ and α_2_ values decreased with Pr_6_O_11_ content. Marginal drop observed in α_2_ value with respect to Pr_6_O_11_ content was attributed to the shifting in voltage at onset of non-linearity. Varistor containing less amount of Pr_6_O_11_ exhibited non-linear characteristic at relatively lower onset voltage than the varistor with high Pr_6_O_11_ content. The *E_b_* varies between 2.8–13.3 V and the value drastically decreased with the Pr_6_O_11_ content. With an average grain size of approximately 5.0 μm, the corresponding *V_gb_* dropped from 0.06 to 0.01 V as the Pr_6_O_11_ content was increased from 0.2 to 0.8 mol %. The range of *V_gb_* was relatively lower than the general *V_gb_* (2–3 V) for high-voltage Pr_6_O_11_ based ZnO varistor reported in [19,20], but in a good agreement with *V_gb_* range for several low-voltage varistor systems developed in [24,25]. The decreasing trend in interface barrier height, *ϕ_B_* was also noted. The value dropped from 0.64 to 0.56. Meanwhile, the *J_L_* value is greatly dependent on the density of ceramic. Ceramic with higher density exhibited lower *J_L_*. Reduction in α and *E_b_* with Pr_6_O_11_ content could be attributed to formation of O vacancies at grain boundaries. It has been well accepted that the oxygen species can increase the density of interface states at grain boundary, thus improve Schottky barrier height [24,26]. The present case suggested that accumulation of excessive Pr depleted the O species and/or promoted the formation of O vacancies in the grain boundary. As a result, the density of interface states reduced and eventually diminished the interface barrier height. This is consistent with the suppression of barrier height at higher Pr_6_O_11_ content and the low O concentration at grain boundary region as observed in EDAX line scanning in Figure 4. In addition, similar observation was reported in the investigation of ZnO/Pr_6_O_11_ thin film interface [21]. It was envisioned that oxygen atom could diffuse from Pr_6_O_11_ layer into ZnO surface during deposition process.

According to this electrical analysis, it seems that the *E_b_* value could be adjusted by controlling the amount of Pr_6_O_11_ in the ceramics. Pr_6_O_11_ has served as the grain boundary activator in these single doped varistor systems. In order to obtain higher non-linearity, additional doping with Cr_2_O_3_, MnO_2_ or CoO is required. The contribution of donor concentration and interface states at grain boundary to non-linearity behaviour of low-voltage Pr_6_O_11_ based ZnO varistors should be established by further detailed studies.

## 3. Experimental Section

### 3.1. Materials

Raw materials were prepared according to the nominal composition of (100 − x) mol % ZnO + x mol % Pr_6_O_11_ where x = 0.2, 0.4 and 0.8. Reagent grade praseodymium (III) acetate hydrate (Pr(CH_2_COOH)_3_·xH_2_O) with the purity of 99.9% (Alfa Aesar) was used as metal salt precursor and citric acid anhydrous (C_6_H_8_O_7_) with the purity of 99.5% (Fluka) was selected as the complexing agent. ZnO powder with the particle size of less than 1 μm and 99.9% purity (Sigma Aldrich) was selected as the host material.

### 3.2. Preparation of Precursor Powder and Ceramics

Uniform coating of Pr citrate gel on ZnO particles was obtained by immersing ZnO powder in bath solution containing citric acid and Pr(III) acetate in deionized water medium for 1 hour retention time at 70–80 °C. Molar ratio of citric acid to Pr acetate was fixed at 3:1 and vigorous stirring was required to improve contact. The mixing process was prolonged for 4 h at 100 °C or until liquid in the mixture dried up. The cake obtained at the end of process was pulverized, sieved and dried at 110 °C for 19 h to produce powder with particle size of less than 100 μm. The dried powder was then calcined at 500 °C for 4 h at heating rate of 3 °C/min. The calcined powder comprising 1.75 wt% polyvinyl alcohol binder was pressed into pellets with 5.0 mm radius and 1.3 mm thickness using Specac Hydraulic Press machine. The pellet was finally sintered at 1200 °C for 1 h in a box furnace (CMTS Model HTS 1400).

### 3.3. Characterizations

TG/DTG analysis was performed using TGA/DSC-1 Mettler Toledo for examining the thermal decomposition profile of precursor powder. Sample was heated from 30–900 °C in air at 10 °C/min. ATR-FTIR spectroscopy (Thermo Nicolet) was utilized to observe the chemical changes on precursor powder before and after calcination process. As-received sample was pressed against germanium crystal plate and analyzed. Phase analysis was conducted on both calcined and sintered samples using XRD (PANalytical (Philips) X’pert Pro PW3040/60) with Cu*Kα* source. Sample was radiated with Ni-filtered Cu*Kα* radiation (*λ* = 1.5428) within the 2*θ* scan range of 20–80°. Surface morphology and elemental analyses of precursor powder and sintered samples were studied under FESEM (JEOL JSM-7200) integrated with EDAX. Sample to view was mounted on Al stub using carbon paint and sputter-coated with gold. Current-voltage (*I-V*) characteristics measurement was carried out on varistor pellets painted with conductive Ag electrode using source measure unit (Keithley 236). Sample was applied with dc voltage from 0 to 100 V in step size of 2.5 V. The non-linearity coefficient, α was determined as d(log *J*)/d(log *E*) where *J* is the current density and *E* is the electrical field (V/mm). Two α values were determined at different *J* regions. α_1_ was measured at low current region which was within the range of 10–100 mA/cm^2^. α_2_ was measured at high current region, within 100–200 mA/cm^2^. The breakdown voltage, *E_b_* was determined as the corresponding *E* at *J* = 1 mA/cm^2^ and the leakage current density, *J_L_* was determined as the corresponding *J* at *E* = 0.8*E_b_*.Voltage per grain boundary, *V_gb_* was calculated as *E_b_(d/D)* where *d* is the average grain size calculated based on line intercept method, *D* is the thickness of the varistor. The interface barrier height, *ϕ_B,_* was estimated according to the following expression:

(4) $$J=AT^{2}\;\text{exp}\left[\frac{\beta E^{1/2}-\phi_{B}}{\kappa T}\right]$$

where *J* is the current density, *E* is the field strength, *κ* is the Boltzmann constant, *A* is the Richardson’s constant (30 Acm^−2^K^−2^) for ZnO, *T* is the absolute temperature, *β* is a constant and is related to the relation as *ϕ_B_* ~ (*rω*)^−1^, where *r* is grains per unit length and *ω* is the barrier width. This method has been applied in [27,28].

## 4. Conclusions

Low-voltage Pr_6_O_11_ doped ZnO varistor ceramics have been successfully prepared by direct modified citrate gel coating technique. Pr citrate gel coating layer was transformed to PrCO_3_OH intermediate and finally converted to nano-scaled Pr_6_O_11_ in two steps of oxidation reaction. Uniform coating of Pr_6_O_11_ on ZnO powder was achieved after calcining at 500 °C for 4 h. The ceramics obtained after sintering at 1200 °C for 1 h had simple microstructure, high density (95.0–98.8%) and a grain size in the range of 4.5–5.2 μm. Preferential segregation of Pr dopants at grain boundaries was improved. Electrical analysis on the Pr_6_O_11_ doped ZnO varistor ceramics demonstrated that the varistor exhibited *E_b_* in the range of 2.8 to 13.3 V. Depending on the Pr_6_O_11_ content, the *V_gb_* decreased from 0.06 to 0.01 V with insignificant alteration in the grain size. The α value in high current region (100–200 mA/cm^2^) ranged from 3.0 to 3.5 and the interface barrier height ranged from 0.56 to 0.64 eV. *E_b_* and α lowering with increasing Pr_6_O_11_ content were associated with reduction in the barrier height caused by variation in O vacancies at grain boundary.

## Figures and Tables

**Table 1:** Microstructure and *I-V* characteristic parameters for Pr_6_O_11_ based ZnO varistor prepared from citrate gel coating technique.

Pr_6_O_11_ (mol %)	*ρ_rel_* (%)	*d* (μm)	α_1_	α_2_	*E_b_* (V/mm)	*V_gb_* (V)	*J_L_* (μA/cm^2^)	*ϕ_B_* (eV)
0.2	98.8	5.2	3.1	3.3	13.3	0.06	695	0.64
0.4	95.0	5.0	2.4	3.5	4.9	0.03	766	0.60
0.8	98.6	4.5	1.8	3.0	2.8	0.01	698	0.56

## References

[1] Levinson L.M., Philipp H.R. (1986). Zinc oxide varistor—A review. J. Am. Ceram. Soc. Bull.

[2] Gupta T.K. (1990). Application of zinc oxide varistor. J. Am. Ceram. Soc.

[3] Pan W.H., Kuo S.T., Tuan W.H., Chen H.R. (2010). Microstructure-property relationships for low voltage varistors. Int. J. Appl. Ceram. Tech.

[4] Clark D.R. (1999). Varistor ceramics. J. Am. Ceram. Soc.

[5] Olsson E., Dunlop G.L. (1989). Characterization of individual interfacial barriers in ZnO varistor material. J. Appl. Phys.

[6] Tao M., Ai B., Dorlanne O., Loubiere A. (1987). Different “single grain junctions” within a ZnO varistor. J. Appl. Phys.

[7] Toplan H.O., Karakas Y. (2002). Grain growth in TiO_2_-added ZnO-Bi_2_O_3_-CoO-MnO ceramics prepared by chemical processing. Ceram. Int.

[8] Hennings D.F.K., Hartung R., Reijnen P.J.L. (1990). Grain size control in low-voltage varistors. J. Am. Ceram. Soc.

[9] Eda K., Inada M., Matsuoka M. (1983). Grain growth control in ZnO varistors using seed grains. J. Appl. Phys.

[10] Souza F.L., Gomes J.W., Bueno P.R., Cassia-Santos M.R., Araujo A.L., Leite E.R., Longo E., Varela J.A. (2003). Effect of addition of ZnO seeds on the electrical properties of ZnO-based varistors. Mater. Chem. Phys.

[11] Kuo S.T., Tuan W.H., Lao Y.W., Wen C.K., Chen H.R., Lee H.Y. (2008). Investigation into the interactions between Bi_2_O_3_-doped ZnO and AgPd electrode. J. Eur. Ceram. Soc.

[12] Wang Q., Qin Y., Xu G.J., Chen L., Li Y., Duan L., Li Z.X., Li Y.L., Cui P. (2008). Low-voltage ZnO varistor fabricated by the solution coating method. Ceram. Int.

[13] Li Y., Li G., Yin Q. (2006). Preparation of ZnO varistors by solution nano-coating technique. Mater. Sci. Eng. B.

[14] Lorenz A., Ott J., Harrer M., Preissner E.A., Whitehead A.H., Schreiber M. (2001). Modified citrate gel techniques to produce ZnO based varistors (Part 1—Microstructure characterisation). J. Electroceram.

[15] Dhage S.R., Pasricha R., Ravi V. (2003). Synthesis of ultrafine TiO_2_ by citrate gel method. Mater. Res. Bull.

[16] Ramirez M.A., Rubio-Marcos F., Fernandez J.F., Lengauer M., Bueno P.R., Longo E, Varela J.A. (2010). Mechanical properties and dimensional effects of ZnO- and SnO_2_-based varistors. J. Am. Ceram. Soc.

[17] Furtado J.G.M., Saléh L.A., Serra E.T., Oliveira G.S.G. (2005). Microstructural evaluation of rare earth-zinc oxide-based varistor ceramic. Mater. Res.

[18] Zhu J.F., Gao J.Q., Wang F., Chen P (2008). Influence of Pr_6_O_11_ on the characteristics and microstructure of zinc varistors. Key Eng. Mater.

[19] Nahm C.W. (2003). Electrical properties and stability of praseodymium oxide-based ZnO varistor ceramics doped with Er_2_O_3_. J. Eur. Ceram. Soc.

[20] Nahm C.W. (2009). The preparation of a ZnO varistor doped with Pr_6_O_11_-CoO-Cr_2_O_3_-Y_2_O_3_-Al_2_O_3_ and its properties. Solid State Commun.

[21] Horio N., Hiramatsu M., Nawata M., Imaeda K., Torii T. (1998). Preparation of zinc oxide/metal oxide multilayered thin films for low-voltage varistors. Vacuum.

[22] Popa M., Kakihana M. (2001). Synthesis and thermoanalytical investigation of an amorphous praseodymium citrate. J. Therm. Anal. Calorim.

[23] Borchert Y., Sonström P., Wilhelm M., Borchert H., Bäumer M. (2008). Nanostructured praseodymium oxide: Preparation, structure and catalytic properties. J. Phys. Chem. C.

[24] Kutty T.R.N., Philip S. (1995). Low voltage varistors based on SrTiO_3_ ceramics. Mater. Sci. Eng. B.

[25] Nakano Y., Ichinose N. (1990). Oxygen adsorption and VDR effects in (Sr, Ca)TiO_3−x_ based ceramics. J. Mater. Res.

[26] Leite E.R., Varela J.A., Longo E. (2005). A new interpretation for the degradation phenomena in ZnO varistor. J. Mater. Sci.

[27] Li C., Wang J., Su W., Chen H., Wang W., Zhuang D. (2001). Investigation of electrical properties of SnO_2_·Co_2_O_3_·Sb_2_O_3_ varistor system. Phys. B.

[28] Lin Y.H., Cai J., Li M., Nan C.W., He J (2008). Grain boundary behavior in varistor-capacitor TiO_2_-rich CaCu_3_Ti_4_O_12_ ceramics. J. Appl. Phys.

